# Daily Intake of a *Phaseolus vulgaris* L. Snack Bar Attenuates Hypertriglyceridemia and Improves Lipid Metabolism-Associated Plasma Proteins in Mexican Women: A Randomized Clinical Trial

**DOI:** 10.3389/fnut.2022.890136

**Published:** 2022-06-03

**Authors:** Aurea K. Ramírez-Jiménez, Ivan Luzardo-Ocampo, M. Liceth Cuellar-Nuñez, Miriam Aracely Anaya-Loyola, Ma. Fabiola León-Galván, Guadalupe Loarca-Piña

**Affiliations:** ^1^Tecnologico de Monterrey, School of Engineering and Science, Monterrey, Mexico; ^2^Research and Graduate Program in Food Science, School of Chemistry, Universidad Autónoma de Querétaro, Queretaro, Mexico; ^3^Instituto de Neurobiología, Universidad Nacional Autónoma de México, Queretaro, Mexico; ^4^Facultad de Medicina, Universidad Autónoma de Querétaro, Querétaro, Mexico; ^5^Facultad de Ciencias Naturales, Universidad Autónoma de Querétaro, Querétaro, Mexico; ^6^Life Science Division, Graduate Program in Biosciences, University of Guanajuato Campus Irapuato-Salamanca, Irapuato, Mexico; ^7^Life Science Division, Food Department, University of Guanajuato Campus Irapuato-Salamanca, Irapuato, Mexico

**Keywords:** oat (*Avena sativa*), dyslipidemia, common bean (*Phaseolus vulgaris* L.), triglycerides, targeted plasma proteomics, microarrays

## Abstract

Current efforts to prevent dyslipidemia are focused on the development of functional products as an alternative for hypertriglyceridemia management. This study assessed the metabolic effect of the daily consumption of a bean and oats snack bar (BOSB) on hypertriglyceridemia biomarkers among Mexican women. An 8-weeks randomized parallel clinical trial (ID: NCT0496694, https://clinicaltrials.gov/ct2/show/NCT04966494) was conducted with 26 hypertriglyceridemic women allocated to BOSB group (TG = 208.18 ± 56.97 mg/dL) and control group (TG = 182.28 ± 51.39 mg/dL). Only the BOSB group consumed 50 g of the product per day. Fasting blood samples were taken from women with an adherence ≥ 90%. A targeted proteomic analysis with plasma samples of control and BOSB groups were conducted using a human obesity antibody array kit and bioinformatic tools provided by the Ingenuity Pathways Analysis (IPA) software. Serum TG levels in the BOSB group decreased by 37.80% (132.04 ± 27.83 mg/dL) compared with the control group (178.87 ± 32.01 mg/dL); glucose levels decreased by 5.69% in the BOSB group (87.55 ± 3.36 mg/dL). A modest body weight (5%) reduction was also found. Forty proteins were differentially modulated by the BOSB consumption (fold change > 1.2). The proteomic analysis revealed the involvement of BOSB bioactives in prevention of monocytes recruitment and localized inflammatory response, inhibition of pre-adipocyte maturation and adipogenesis, inhibition of hepatic b-oxidation, and potential satiety regulation. These results are promising since the mere intervention with the BOSB reduced serum TG without diet restriction, giving insights for further research in prevention of hypertriglyceridemia.

## Introduction

Non-communicable diseases (NCDs) are among the main causes of death globally, especially diabetes, obesity, and cardiovascular disease, which are highly prevalent in developing and developed countries (1). Hypertriglyceridemia is a serious condition worldwide considered an important risk factor for developing NCDs, especially cardiovascular diseases (2). Hypertriglyceridemia is defined as the presence of blood triglycerides > 150 mg/dL, low-density lipoproteins < 130 mg/dL and total cholesterol < 200 mg/dL (3). The high intake of dietary lipids and rapidly digestible carbohydrates are one of the main causes of hypertriglyceridemia. Other possible factors influencing the development of this disorder are low triglycerides (TG) clearance rate, insulin resistance and obesity, and lipoprotein lipase dysfunction (4). Since the diet is a strong factor influencing triglyceride accumulation, dietetic management is the logical approach to tackle hypertriglyceridemia.

Common beans (*Phaseolus vulgaris* L.) are considered an important source of phytochemicals with hypolipidemic activity such as: dietary fiber, oligosaccharides, resistant starch, and phenolic compounds are the main components in beans with reported TG lowering properties (5). Several mechanisms have been proposed to explain this effect, for example, the promotion of satiety neuropeptides *via* activation of FFAR by dietary fiber metabolites in the large intestine, increased clearance rate, higher expression of hepatic low-density lipoprotein receptor (LDLR), and modulation of the lipogenic enzymes (Fatty acid synthase, FAS; and Acetyl CoA-Carboxylase, ACC) (6). Studies using animal models have demonstrated that consumption of cooked beans decreases circulating triglycerides and visceral adiposity (7, 8), total cholesterol (9), and induce the expression of CPT1, a transcription factor that stimulates β-oxidation (6). On the other hand, it has also reported the effect of consuming a baked corn/bean snack on lowered serum lipids and differentiated liver gene expression in C57BL/6 mice fed a high-fat diet by inhibiting PPARγ and SREBF2 (10). Nevertheless, there are reports of the null effect of beans (navy beans) on TGs when consumed in combination with edible plant extracts (11) or consumed as part of snack formulation (banana muffin and pineapple pie) by hypercholesterolemic children (12).

Despite these benefits, common beans consumption has decreased in the last years due to the intestinal discomfort that some people may experience, and the long processing times required for cooking. In this sense, the functional foods market, especially the ready-to-eat sector, has gained interest due to the convenience and health benefits offered to consumers. Snack bars are popular products in the health sector due to its convenience to incorporate phytochemicals with known bioactivities, such as common beans. In previous work, we reported the promising potential of a baked beans-oats snack bar (BOSB) as a functional product due to outstanding levels of dietary fiber (32.46%), resistant starch (8.45%), and total flavonoids (5.64 mg rutin equivalent/g) (13). Therefore, we conducted a clinical trial in a Mexican women group to assess the metabolic effect of the daily BOSB consumption on hypertriglyceridemia biomarkers.

## Materials and Methods

### Recruitment

**FIGURE 1:**
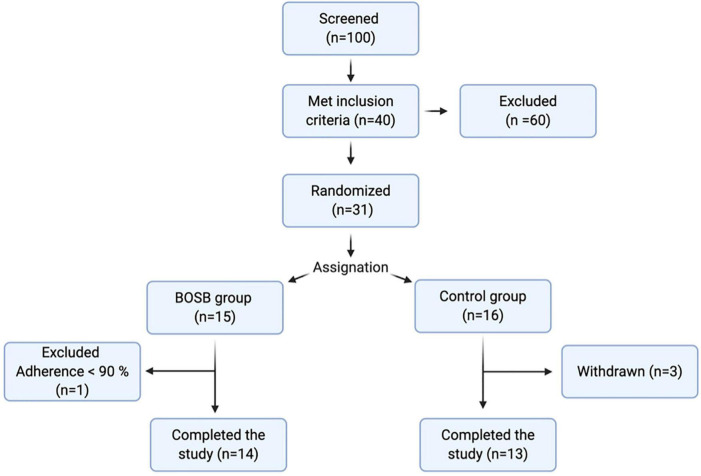
Study design. Flow chart for overall screening procedure.

This protocol followed the guidelines of the Declaration of Helsinki and was approved by the Internal Ethics Committee for Human Research of the Natural Sciences Faculty at Universidad Autonoma de Queretaro. All women received oral and written information regarding all aspects of the study, voluntarily accepted to participate (if meeting inclusion criteria), and signed consent forms. Women were recruited by various means: informative flyers in hospitals, the invitation to parents during health campaigns in schools of the area, announcements on college radio. Women aged 18–45 years that met the inclusion criteria were enrolled: no current diagnosed illness, triglyceride levels between 150 and 350 mg/dL (measured in the first appointment), no allergies to common beans or derived products, not pregnant or lactating. Subjects were asked to fill a clinical record and eating habits and physical activity questionnaire. Participants were excluded if they had current diagnosed diabetes, hypertension, irritable bowel syndrome, or cancer; were taking any pharmacological treatment including anti-inflammatory and lipid-lowering drugs, had fasting glucose > 100 mg/dL, total cholesterol < 240 mg/dL, LDL-C > 160 mg/dL, or reported intolerance/allergies to the snack bar ingredients. From the 100 women initially screened, 40 met all inclusion criteria; 9 of these withdrew the study before allocation, three during the study, and one was eliminated due to the low adherence to the treatment (Figure 1).

### Study Design

The study was a parallel, randomized intervention trial. Subjects were assigned to one of two groups, the BOSB group that consumed 50 g of the product for 8 weeks (25 g two times per day) or a control group with no intake of BOSB. Both groups received nutritional orientation according to the guidelines of the Official Mexican Regulation NOM-043-SSA2-2005. During this session, women were instructed on general nutritional guidelines and meal plans to follow during the study that are recommended for hypertriglyceridemia in people. A food frequency questionnaire was performed at baseline and after finishing the trial (14) in order to know the calorie intake for each participant. For the food frequency questionnaire, we used the electronic version of the questionnaire validated by Galván-Portillo et al. (14). For this purpose, women went to the Nutrition Clinic of the Natural Sciences Faculty (NCNSF) at Universidad Autonoma de Queretaro to take the questionnaire aided by the Clinic personnel.

BOSB were provided to participants on a weekly basis, furthermore, participants were asked to complete a treatment adherence format that was collected weekly; the empty packages of the BOSB consumed were also collected to monitor adherence (determined as percentage of the total expected consumed packages over a week). During this meeting, weight, waist and hip circumference, and body fat percentage were monitored (Omron bioimpedance equipment, model Hbf 501). Once the trial began, elimination criteria included: adherence < 90%, illness or medication during the study, presence of acute inflammation, or voluntary withdrawal.

Snack bars were prepared based on the following formulation proposed by Ramírez-Jiménez et al. (13): cooked bean flour (30%), oat flour (26%), emulsifier (1%), and corn oil (3%). Packages contained 50 g of the product (6 pieces), each package was labeled with the product batch number, elaboration and expiration date, and the adequate quantity of product was delivered weekly to the participants.

### Anthropometry

Anthropometric measurements were done according to the World Health Organization (WHO) guidelines. Women were instructed to fast overnight (12 h) until arrival at the NSF, where a trained dietetic practitioner recorded waist and hip circumference. Weight (kg), body mass index (BMI), body fat (%), visceral fat (%) and lean mass (%) with a body composition analyzer X-Scan plus II (iCare Co., Ltd., South Korea).

### Sample Collection and Clinical Markers

At baseline and at the end of the study, fasting venous blood was collected by intravenous cannula in Vacutainer tubes (EDTA and tubes without anticoagulant). Tubes without anticoagulant were used for the further separation of serum, which was subjected to analysis of fasting glucose, TGs, total cholesterol, LDL, HDL using Spinreact kits (Spinreact S.A., Girona, Spain) and a Mindray BS-120 analyzer (DS, US.). EDTA tubes were used for plasma isolation to obtain the proteomic markers profile. All the serum/plasma samples were immediately processed or aliquoted and frozen at −70°C with liquid nitrogen. Plasma measurements included: High-sensitive C-reactive protein (hs-CRP) determined by the Accubind kit (Monobind Inc., Lake Forest, United States), insulin using the Accubind rapid Insulin Test (Monobind Inc., Lake Forest, United States) and insulin resistant by the HOMA index [HOMA = (glucose × insulin)/405].

### Targeted Proteomics

A plasma protein screening was performed to detect differentially expressed cytokines implicated in obesity and inflammation pathways. Plasma samples from the BOSB and control group were pooled (randomly assigned) in four subsets for each group, and then analyzed for differential expression of 182 proteins using a Label-based Human Obesity Antibody Array kit (AAH-BLG-ADI-1-4 microarray, RayBiotech, Norcross, GA, United States) according to the manufacturer’s recommendations. Microarray slides were scanned with a GenePix 4100A laser scanner (Axon Instruments, Sunnyvale, CA, United States), with laser excitation at 532 nm, 800 PMT gain, and 332 V. Images were acquired with the GeneGenepix Pro 6.0.1.27 software. Functional annotation was performed with DAVID Bioinformatics Resources 6.8

### Statistical Analysis

Results are presented as mean ± standard deviation (SD) when normally distributed and as the mean and confidence interval (LCL, UCL, CI95%). Differences between groups at baseline were compared using Student *t*-test. An analysis of covariance (ANCOVA) was performed in order to compare the test (BOSB) and the control group with baseline values for BMI as covariates. A *p*-value < 0.05 was considered statistically significant. Statistical analyses were performed using JMP Software (Version 14).

For microarrays analysis, normalization was carried out using the median intensity values (from which background signal was subtracted) with the Analysis Tool Software for RayBio^®^ Human 55 Biotin-Label Based Obesity Antibody Arrays (Human L-182 Array, Glass Slide, RayBiotech, Inc., Norcross, GA, United States). Normalized data were log2-transformed to calculate the fold-change for each protein, a value > 1.2 was considered as a differential expression. Data were interpreted using Ingenuity Pathway Analysis (IPA) v 9.0 (Ingenuity Systems, Inc., Redwood City, CA, United States) to identify biological processes and functions modulated by the BOSB consumption.

## Results

### BOSB Proximal and Nutraceutical Composition

The BOSB proximal and nutraceutical composition is shown in Table 1. The BOSB had 19.25% protein, and 10% lipids. Total dietary fiber was the major component within carbohydrates (50.15 ± 0.37%), moreover, resistant starch (1.75%), and two oligosaccharides were quantified (raffinose and stachyose). The amount of condensed tannins was significantly higher (*p* < *0.05*) than total flavonoids. Three phenolic acids (gallic, chlorogenic, and ferulic acids) and three flavonoids [(+)-catechin, rutin, and quercetin] were identified by HPLC-DAD, being gallic acid (237.97 ± 48.50 mg/g), chlorogenic acid (370.35 ± 38.99 mg/g), and (+)-catechin (82.31 ± 15.16) the most abundant metabolites.

**TABLE 1:** Chemical and nutraceutical composition of BOSB. **(A)** Proximal composition, oligosaccharides, total phenolic compounds, and antioxidant capacity; **(B)** Free phenolic compounds quantified by HPLC-DAD.

(A)		

Components	BOSB	
**Proximal composition (%)**		
Protein	19.25 ± 0.20	
Lipids	10.05 ± 0.23	
Ash	1.42 ± 0.03	
Moisture	3.28 ± 0.05	
Carbohydrates	60.57 ± 0.37	
Total dietary fiber	30.38 ± 0.41	
Insoluble dietary fiber	22.67 ± 0.25	
Soluble dietary fiber	7.70 ± 0.66	
**Oligosaccharides by HPLC-RID (mg/g)**		
Raffinose	8.22 ± 0.61	
Stachyose	25.34 ± 1.45	
**Total phenolic compounds**		
Total flavonoids (mg RE/g)	8.40 ± 0.01	
Condensed tannins (mg CE/g)	12.46 ± 0.42	
**Antioxidant capacity (TEAC, mmol/g)**		
DPPH	6.17 ± 0.21	
ABTS	9.54 ± 0.13	

**(B)**		

**Phenolic compound**	**RT (min)**	**Amount (μg/g)**

**Phenolic acids**		
Gallic acid	1.71	237.97 ± 48.50
Chlorogenic acid	2.04	370.35 ± 38.99
Ferulic acid	5.82	2.18 ± 1.31
**Flavonoids**		
(+)-Catechin	2.29	82.31 ± 15.16
Rutin	3.21	3.91 ± 0.22
Quercetin	13.39	17.37 ± 4.73

*Results are the mean ± SD of three independent experiments in triplicates. The dietary fiber content and its fractions (insoluble and soluble) were expressed relative to the carbohydrate content. All results from the proximal analysis were expressed in dry basis. For the HPLC-DAD quantification of phenolics, each phenolic was expressed in micrograms equivalents of each phenolic compound/g sample and the results are the mean ± SD of four different BOSB batches, in triplicates. ABTS: 2,2′-azino-bis (3-ethylbenzothiazoline-6-sulfonic acid); CE: (+)-catechin equivalents; DPPH: 2,2-diphenyl-1-picrylhydrazyl; RE: Rutin equivalents; TEAC: Trolox equivalent antioxidant capacity.*

### Study Design and Baseline Measurements

Twenty-six women met all inclusion criteria and completed the study with adequate adherence (> 90%) to the dietary intervention (Figure 1). Baseline characteristics of the 26 subjects allocated to study are shown in Table 2. The mean age was 34.5 years, while the mean triglyceride levels were 204.47 ± 57.22 and 179.90 ± 30.61 mg/dL. Control and BOSB groups were comparable in age, weight, height, BMI, waist and hip circumference, and energy intake. At baseline the calorie intake was similar in both groups, 2,547.66 ± 739.82 and 2,521.23 ± 623.25 Kcal for BOSB and control group, respectively. In both cases, energy intake exceeded the 2,000 kcal/day recommended for women of the aforementioned characteristics. The mean baseline BMI was ∼ 30.4 which meets the criteria for obesity class I (WHO, 2004). No significant differences were observed in biochemical markers between groups (*p* > 0.05).

**TABLE 2:** Baseline characteristics of participants.

Characteristic	BOSB group (*n* = 14)	Control group (*n* = 12)
Age (y)	38.00 ± 7.39	33.00 ± 8.30
Weight (Kg)	73.79 ± 10.77	71.76 ± 14.57
Height (cm)	155.49 ± 5.46	154.95 ± 7.65
BMI ^1^	30.59 ± 4.84	29.93 ± 6.19
Waist circumference (cm)	90.41 ± 9.30	87.66 ± 10.80
Hip circumference (cm)	108.75 ± 8.61	104.71 ± 9.99
Body fat (%)	38.75 ± 4.28	37.48 ± 4.22
Energy intake (kcal/d)	2,547.66 ± 739.82	2,521.23 ± 623.25
Glucose (mg/dL)	92.83 ± 9.58	87.22 ± 7.68
Total cholesterol (mg/dL)	187.42 ± 33.05	179.90 ± 30.61
Triglycerides (mg/dL)	204.47 ± 57.22	174.72 ± 48.38
HDL^2^ (mg/dL)	53.96 ± 8.95	53.64 ± 8.61
LDL^3^ (mg/dL)	92.56 ± 30.21	91.32 ± 26.96
hs C-reactive protein (μg/ml)	6.33 ± 4.39	4.02 ± 3.40
Insulin (mUI/ml)	14.55 ± 10.39	13.06 ± 4.80
HOMA index	3.40 ± 2.72	2.80 ± 1.10

*^1^Body mass index (25–29.9 overweight, > 30 obesity).*

*^2^High-density lipoproteins (Normal values: HDL > 40 mg/dL).*

*^3^Low-density lipoproteins (Normal values: LDL < 130 mg/dL; high values: 150–159 mg/dL; very high values LDL > 160 mg/dL).*

*Results are expressed as the mean ± SD. A paired t test was used to compare the baseline features. Compared with the corresponding baseline value (p < 0.05), there were no significant differences in baseline features between groups.*

### Effect of BOSB Consumption on Anthropometric Measurements

Table 3 shows the anthropometric measurements and biochemical markers before and after BOSB consumption. Weight, BMI, waist and hip circumference as well as body fat were not significantly different before and after the treatment between groups (*p* > 0.05). Energy intake per day tended to decrease after the BOSB intervention, however, this reduction was not significant when compared with the control group.

**TABLE 3:** Changes in anthropometric measurements after 8-week consumption of BOSB.

Outcomes	BOSB group (*n* = 14)	Control group (*n* = 12)	*p* value	Difference Control vs BOSB
**Weight (Kg)**				
Baseline	73.79 (52.80, 93.50)	71.76 (52.30, 97.40)		
Final value	71.48 (70.37, 72.58)	71.87 (70.69, 73.06)	0.6215	0.39 (−1.23, 2.01)
Change	−2.31	0.11		
**Body mass index (BMI)**				
Baseline	30.59 (21.00, 40.50)	29.93 (22.50, 44.40)		
Final value	29.76 (29.31, 30.21)	29.93 (29.44, 30.42)	0.5932	0.17 (−0.49, 0.83)
Change	−0.83	0		
**Waist circumference (cm)**				
Baseline	90.41 (70.20, 102.70)	87.66 (73.50, 106.85)		
Final value	87.74 (85.97, 89.51)	87.53 (85.59, 89.49)	0.8737	0.20 (−2.42, 2.83)
Change	−2.67	−0.13		
**Hip circumference (cm)**				
Baseline	108.08 (89.80, 120.50)	104.71 (91.80, 122.9)		
Final value	105.29 (103.26, 107.31)	104.50 (102.30, 106.70)	0.5883	0.79 (−2.20, 3.78)
Change	−2.79	−0.21		
**Body fat (%)**				
Baseline	38.75 (29.90, 45.30)	37.48 (32.00, 46.60)		
Final value	38.09 (37.32, 38.86)	37.81 (36.88, 38.74)	0.6303	0.28 (−0.92, 1.49)
Change	−0.66	0.33		
**Energy intake (Kcal/d)**				
Baseline	2,547.66 (1,097.0, 37,650.50)	2,521.23 (1,783.60, 3,906.9)		
Final value	2,065.06 (1,645.70, 2,484.42)	2,297.20 (1,793.66, 2,800.73)	0.4663	232.14 (−423.16, 887.44)
Change	−434.33	−285.63		

*No significant differences were found between BOSB and control for any variable. Values represent the mean and 95% confidence interval (CI95%) in parentheses. One-way ANOVA was used for analyzing the outcomes, means were contrasted using a paired t test (p < 0.05).*

### Effect of BOSB Consumption on Glucose and Lipid Profile

**FIGURE 2:**
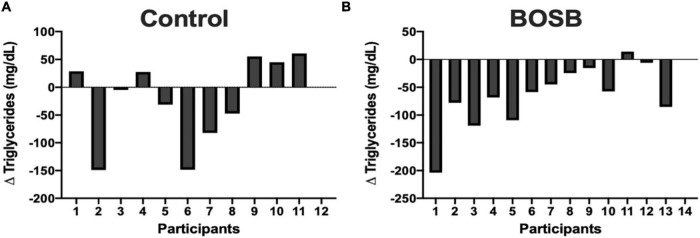
Individual triglyceride differences in **(A)** Control group and **(B)** BOSB group after 8-weeks consumption.

Among biochemical markers (Table 4) serum TG levels significantly (*p* = 0.0324) decreased by 37.80% (132.04 ± 27.83 mg/dL) in the BOSB group compared with the control group that slightly increased after the study (178.87 ± 32.01 mg/dL). The intragroup variability for TG was higher in the control group, as shown in Figure 2. Fifty percent of participants showed reductions of plasma TG in the control group, whereas > 85% of participants that consumed the BOSB reduced their TG levels after the intervention. Although no statistical significance was found, glucose levels also decreased by 5.69% in the BOSB group (87.55 ± 3.36 mg/dL), while the control group increased by 4.41% (91.07 ± 3.62 mg/dL). Similar reductions were observed for body weight (−5%) and energy intake (−18.94%) in the BOSB group. Total cholesterol, HDL, and LDL levels did not show significant differences between groups (*p* > 0.05), even though plasma concentrations were higher at the end in the control groups. For baseline and final values, only TG in the BOSB group and HDL in both groups were statistically different, with slightly higher values.

**TABLE 4:** Changes in biochemical markers after 8-week consumption of BOSB.

Outcomes	BOSB group (*n* = 14)	Control group (*n* = 12)	*p* value	Difference Control vs BOSB
**Glucose (mg/dL)**				
Baseline	92.83 (76.10, 110.60)	87.22 (73.10, 100.40)		
Final value	87.55 (84.19, 90.91)	91.07 (87.45, 94.69)	0.1524	3.52 (−1.42, 8.46)
Change	−5.28	3.85		
**Cholesterol (mg/dL)**				
Baseline	187.42 (147.05, 248.15)	179.90 (125.50, 233.20)		
Final value	181.86 (172.32, 191.41)	192.20 (182.29, 202.12)	0.1466	10.34 (−3.42, 24.10)
Change	−5.56	12.30		
**Triglycerides (mg/dL)**				
Baseline	204.47 (141.30, 307.25)	174.72 (74.85, 261.15)		
Final value	132.04 (104.21, 159.87)**	178.87 (146.86, 210.88)	0.0324	46.83 (4.41, 89.25) *
Change	−72.43	4.15		
**HDL (mg/dL)**				
Baseline	53.96 (32.30, 66.10)	53.64 (39.40, 67.40)		
Final value	63.41 (58.66, 68.17)**	64.80 (59.85, 69.76)**	0.6761	1.39 (−5.47, 8.25)
Change	9.45	11.16		
**LDL (mg/dL)**				
Baseline	92.56 (45.62, 135.54)	91.32 (49.98, 146.46)		
Final value	92.75 (85.34, 100.17)	93.48 (85.77, 101.19)	0.888	0.73 (−9.96, 11.42)
Change	0.19	2.16		
**hs C-reactive protein (μg/ml)**				
Baseline	6.33 (1.03, 12.57)	4.02 (0.68, 12.68)		
Final value	3.86 (0.72, 7.01)	4.50 (1.28, 7.73)	0.7695	0.64 (−3.87, 5.14)
Change	−2.47	0.48		
**Insulin (mUI/ml)**				
Baseline	14.55 (4.97, 37.93)	13.06 (7.60, 23.27)		
Final value	12.35 (10.88, 13.82)	10.64 (9.17, 12.12)	0.0976	1.71 (−0.37, 3.79)
Change	−2.20	−2.42		
**HOMA index**				
Baseline	3.40 (0.95, 9.66)	2.80 (1.47, 4.99)		
Final value	2.67 (2.30, 3.04)	2.46 (2.08, 2.84)	0.3981	0.21 (−0.31, 0.73)
Change	−0.73	−0.34		

*Denotes statistical difference between treatments (p < 0.05). **Denotes statistical difference between baseline and final values. Values represent the mean and 95% confidence interval (CI95%) in parentheses. One way ANOVA was used for analyzing the outcomes, means were contrasted using a paired t test (p < 0.05).

### Effect of BOSB Consumption on Inflammation and Diabetes Markers

The inflammatory status of the women was measured using the hs-CRP test, this is a validated low-grade chronic inflammatory marker that is related to dyslipidemia and cardiovascular disease. At baseline, hs-CRP values were 6.33 ± 4.39 and 4.02 ± 3.40 μg/ml for BOSB and control group; after the 8-weeks intervention, there were no significant changes (*p* = 0.888) between groups. Noticeably, for the BOSB group, hs-CRP decreased 39% compared with a 7.46% increase in the control group.

### Microarray Analysis

**FIGURE 3:**
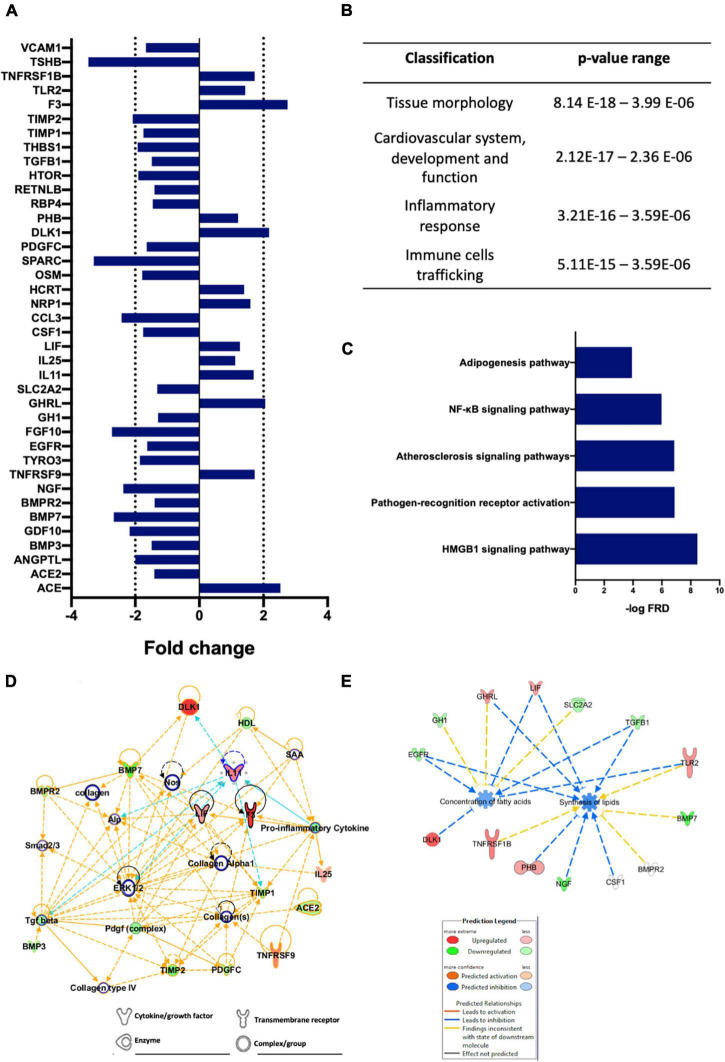
Differential expression of plasma proteins and downstream analysis showing the main regulated pathways after BOSB consumption. **(A)** Fold-changes of the modulated plasmatic proteins after the BOSB consumption; **(B)** Top morphological functions; **(C)** Top regulated canonical pathways; **(D)** IPA protein network associating cytokine/growth factors, enzymes, transmembrane receptors, and complexes; **(E)** Main regulated pathways linked to the synthesis of lipids.

Figure 3 shows the observed changes in plasma proteins after the BOSB consumption. Out of 182 analyzed proteins, 40 were differentially modulated by BOSB [fold change (FC) > 1.2] (Figure 3A). TSH, Osteonectin, FGF-10, BMP-7, MIP-1a, b-NGF, BMP-3b/GDF-10, TIMP-2, and the Ang-like factor were the top downregulated proteins (fold-change > 2), whereas Ghrelin, Pref-1, ACE/CD43, Tissue Factor (CD142) were the top upregulated proteins (fold-change > 2). Moreover, the adhesion molecule VCAM1 (FC: −1.67), the cardiovascular marker ACE-2 (FC: −1.41), and the inflammatory Resistin-like molecule β (FC: −1.4) were also downregulated. Further details are shown in Supplementary Table 1. Functional annotation showed that the regulated proteins were involved in canonical pathways related to tissue morphology, cardiovascular system, inflammatory response and immune cells trafficking (Figure 3B). Furthermore, the predictive analysis with IPA indicated that HMGB1 and the pathogen-recognition receptor activation signaling were the top canonical pathways modulated by the protein arrangement (Figure 3C). A complete description of proteins matching the most relevant canonical pathways and morphological functions are depicted in Supplementary Table 2. A modulatory effect was observed in metabolic pathways related to the cardiovascular system and the inflammatory process (Figure 3D). From this network, we observed the downregulation of TGFβ, the BMP’s family, ACE2 among others. Finally, predicted downstream protein interactions associated to the concentration of fatty acids and the synthesis of lipids are shown in Figure 3E. Upregulation of Pref-1 (DLK1) and LIF leads to inhibition of processes involved in the regulation of fatty acids concentration, while involvement of Prohibitin (PHB) upregulation and TGF-b downregulation inhibits biosynthesis of lipids.

**FIGURE 4:**
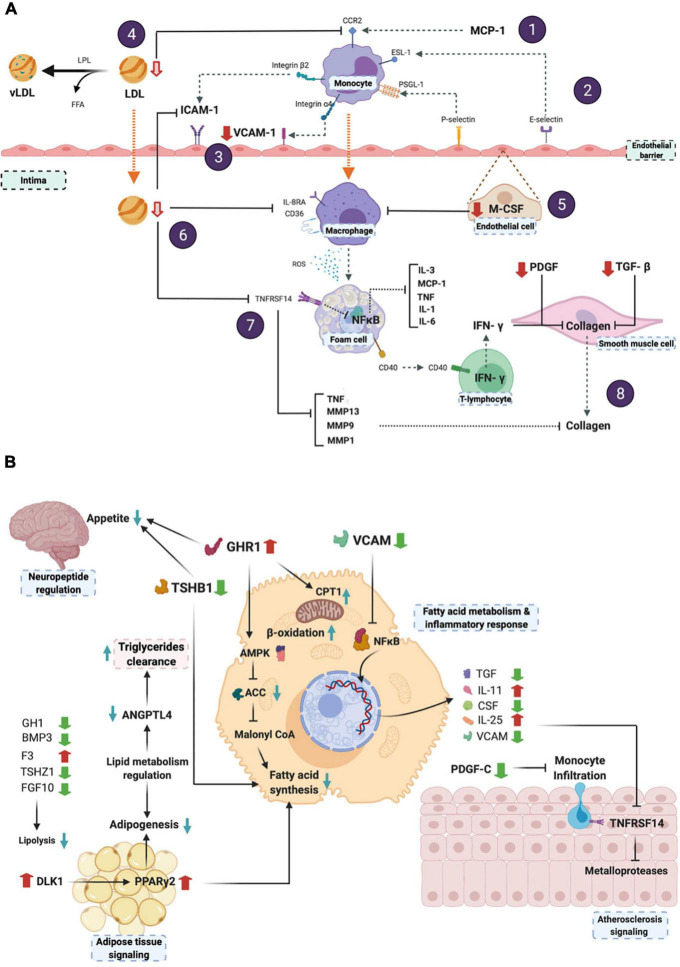
**(A)** Predicted effects of BOSB consumption on atherosclerosis-related metabolic pathways; **(B)** Integration of the main BOSB-regulated systemic pathways associated to neuropeptide regulation, lipid metabolism and atherosclerosis signaling. This figure was generated with BioRender.com. (1) The atherosclerosis pathway initiates with LDL deposition and monocyte recruitment to the endothelial barrier mediated by MCP-1 and CCR2. (2) After monocyte rolling mediated by E-selectin and receptor ESL-1, (3) the lower levels of VCAM-1 found in the microarray analysis, and predicted reductions in plasma LDL levels, reduce endothelial cell adhesion. (4) Lower predicted LDL also inhibits CCR-2 expression and MCP-1 mediated *trans* endothelial migration of monocytes into the intima. It is known that ROS and oxidized-LDL uptake *via* scavenger receptors leads to lipid accumulation and foam cell formation that secrete a number of inflammatory cytokines. (5) Reduced M-CSF levels inhibit monocyte-to-macrophage differentiation, (6) as well as a decreased LDL migration into the intima and inhibition of CD36/IL-8RA. (7) This has a predicted effect on TNFRSF14 which may inhibit the NF-kB pathway and the downstream production of inflammatory cytokines (IL-3, MCP-1, TNF, IL-1, IL6), as well as TNF and matrix metalloproteases, involved also in inflammation processes and in atherosclerosis onset. (8) Finally, reduction observed for PDGF and TGF-β inhibits exacerbated collagen production *via* the CD40 receptor found in the surface of foam cells and T-lymphocytes, that may act *via* IFN-γ. Collagens constitute a component of the extracellular matrix in the atherosclerotic plaque.

Figure 4A shows the predictive effect of the differentially expressed proteins on the atherosclerosis signaling pathway, whereas Figure 4B depicts the metabolic integration of the potential effects of the 8-week BOSB consumption. As observed in Figure 4A, certain processes such as endothelial adhesion, (1, 2) monocyte migration (3, 4), and macrophage differentiation (5–7) are predictively inhibited by BOSB consumption. This leads to lower cytokines production that may inhibit the NF-kB pathway, and the atherosclerotic process (7, 8).

## Discussion

The development of NCDs such as hypertriglyceridemia is strongly associated with consumption of fatty foods, fast food, and soft drinks (15). As snacks might significantly contribute to the daily energy intake (16), the development of healthy alternatives incorporating functional ingredients such as oats and common beans might reduce the risk of cardiovascular conditions, providing bioactive compounds with potential health benefits. The BOSB provides higher protein (+52.41–54.49%) and lower lipid (−50%) content than previously assayed oat and beans formulations (13) or pulse-based oat bars (17). In this work, we found that the TDF concentration was also higher than that reported for oat-common bean cookies (+ 93.37%) (18). The insoluble to soluble fiber ratio was 3:1, similar to the value reported by Morales-Polanco et al. (19) for crackers made with 80% dehulled oats and added with 20% pea flour (∼3.3:1). A higher insoluble fiber ratio not only improves texture, but also may have an effect on carbohydrates digestibility, decreasing the glycemic index (20). Moreover, the BOSB has a significant oligosaccharide content, composed of raffinose and stachyose, as well as resistant starch (Table 1), that have been shown to decrease serum triglycerides and high-density lipoprotein cholesterol (HDL-C) (21). It is worth to note the antioxidant capacity of the snack, which may be exerted by gallic and chlorogenic acids, as well as the flavonoid (+)-catechin (Table 1). As reported in our previous work (13), most of the protein and antioxidant content comes from common beans, a suitable strategy for improving the chemical and nutraceutical properties of snacks (13, 22, 23). Moreover, some of the reported phenolics in this study have been largely associated to several lipid-lowering mechanisms (5).

Despite the no significant changes on anthropometry and body composition, we observed that weight and body fat had a tendency toward reduction in the BOSB group (Table 2). These findings, although not statistically significant, could have a possible physiological relevance. Nonetheless, one reason that could explain the lack of change is the time of intervention, which may not have been enough to show differences. It has been reported in other clinical studies that the administration of supplements has had effects from the fourth week (24). It should be considered that most of the works published in this area use purified extracts or concentrates of the compound of interest. For example, obese subjects consumed 445 mg of an encapsulated extract of *P. vulgaris*, and the diet was controlled to consumed 2,000 to 2,200 Kcal/day. After 30 days of supplementation, weight, BMI, fat, and waist/hip changes were observed (24). A similar study reported that the regular intake (2,400 mg/day) for 35 days of white kidney beans extract (*Phaseolus vulgaris L*.) induced weight loss compared with placebo in obese human subjects (25). In the present study, BOSB was provided as a substitute for highly caloric foods commonly consumed as snack. The diet was not controlled since the bar would be consumed as a convenience food and not as a drug or food supplement.

Regarding the biochemical markers, an increase in HDL levels was observed in control (64.80 mg/dL) and BOSB (63.41 mg/dL) groups compared to the initial conditions. However, there was no difference between both groups (Table 4). This might be influenced by the dietary orientation offered to participants prior to the study, which probably had a positive effect on habits for polyunsaturated fats intake. One of the most important findings was the reduced TG levels after BOBS consumption. The reduction found for the BOSB group was 35.43% (from 204.47 mg/dL to 132.04 mg/dL), while in the control group the TG levels increased 2.37% (from 174.72 mg/dL to 178.87 mg dL) at the end of the study. Although the literature regarding the effect of beans on TG in clinical studies is limited, a decrease in serum TG was observed after 8-weeks intake of a white kidney bean extract (26). It is known that some bioactive compounds in beans could modulate transcription factors, and attenuate TG levels. For instance, chlorogenic acid and (+) catechin, two of the most abundant phenolic compounds found in BOSB, have been associated with decreased serum lipid levels by modulating PPARα and LXRα transcription (27). Moreover, the non-digestible fraction of beans, phytosterols, and saponins exert hypolipidemic effects due to a reduced absorption of lipids at intestinal level by binding of bile acids, cholesterol micelles disruption, and downregulation of lipogenic proteins *via* the liver X receptor pathway (5).

Glucose had a slight reduction for the BOSB group (−5.69%), whereas it increased in the control group (+4.41%). There is evidence that the consumption of different bean varieties induces a low glycemic response and improves glucose control in patients with diabetes (28, 29). Since BOSB had a medium GI value (GI = 52, data not shown) very close to a low glycemic value, it was reasonably expected that the glucose load would improve. Supporting our findings, a meta-analysis showed that the legume consumption improves glycemic control markers, including fasting glucose, glycosylated hemoglobin (HbA1c) and fructosamine (30). Even in patients with type II diabetes, including *P. vulgaris* only to the diet can improve blood glucose levels (30). This decrease has been associated with the content of resistant starch and slow-digesting carbohydrates by several mechanisms. For instance, dietary fiber could trap nutrients, such as glucose or cholesterol, reducing their absorption in the intestinal tract. Other mechanisms of action include binding of bile acids, increase of fecal cholesterol excretion and a putative effect on hepatic low-density lipoproteins receptor for improved lipoproteins clearance (5). Besides, BOSB contains a significant percentage of oats (26%), that include β-glucans that have been recognized as cardioprotective agents by the FDA. The viscosity of these compounds in solution decreases absorption of glucose and lipids. In general, viscous fibers have been shown to reduce postprandial response more effectively than non-viscous fibers (31).

The BOSB consumption displayed a significant modulation of proteins linked to inflammation-associated response, cardiovascular disease, and adipogenesis. The most significant pathway predicted by the IPA analysis was the high-mobility group box 1 protein (HMGB1), suggesting that BOSB consumption has influence on the immune response and cytokine signaling (Figure 3). HMGB1 activates several receptors such as the advanced glycation-end products receptor (RAGES) and the toll-like receptor 2 (TLR2), this latter, highlighted as the second most potentially regulated pathway (Figure 3A). The likely effect of BOSB, might be reflected on inhibition of cell migration, increased production of adhesion molecules, and the release of cytokines such as TNF-α, IL-1α, IL-1β, IL-Ra, IL-6, IL-8, and MCP-1 (32).

A remarkable finding, was the upregulation of the protein delta homolog 1 (DLK1), which is of great interest since it has been shown to inhibit adipogenesis and increase the expression of PPARs, thus proposed as therapeutic target for the treatment of obesity and diabetes (33). The overall dominance of inhibitory mechanisms in the fatty acid synthesis and concentration seems to be related to the DLK1 increase and to a higher concentration of GHR1, involved in appetite increase, but mainly on the starvation state (34). Reports have linked GHR1, independently of its orexigenic effect, with an anti-obesogenic effect due to the inhibition of lipogenic enzymes such as acetyl CoA-carboxylase and fatty acid synthase (FAS) (35).

Results also showed a decrease in TGF-β, a protein superfamily included in the bone morphogenetic proteins (BMP) group, expressed in endothelial cells, smooth muscular cells, macrophages, and T cells in atherosclerotic lesions (36). Although ACE and ACE2 proteins showed opposite behavior, ACE2 has a primary biological importance due to its catalytic activity in the angiotensin conversion, thus, reducing its activity along with VCAM1 reduction and IL-11 increase, which has a potential antihypertensive effect (37). Since IL-11 is an inhibitory factor of adipogenesis by inhibition of the F2α-mediated pre-adipocyte differentiation (38), it could be inferred that the BOSB bioactives potentially suppressed these mechanisms. For instance, stachyose, the most abundant oligosaccharide in BOSB, has shown ameliorative effects on long-term high-fat diet (HFD) *in vivo* upregulating TNF-α levels and increasing peripheral blood leukocytes populations to alleviate the HFD-induced colon and liver inflammation (39).

According to the downstream analysis with IPA (Figure 4A), atherosclerosis-related pathways were modulated by the BOSB intake. The predicted blockade of the interaction between MCP-1 and CCR2 avoids monocyte recruitment and endothelial extravasation through the endothelial barrier, reducing the expression of adhesion molecules and a higher development of local inflammation (40). Furthermore, the predicted reduction of LDL inhibits C-C chemokine receptor type 2 (CCR2), targeted by the monocyte chemoattractant protein-1 (MCP-1), which is potentially hindered, blocking the *trans* endothelial migration of monocytes into the intima. Moreover, BOSB intake promotes oxidized LDL uptake, participating in potential lipid accumulation and foam cell formation (3). A reduced macrophage trafficking into the intima avoids the production of pro-inflammatory factors, obstructing the amplification of the local inflammatory response (4).

Regarding Figure 4B, bioactive compounds from BOSB not only participates in atherosclerosis pathways, but these are also involved in the hepatic immune and inflammatory response, obstructing NF-κB translocation into the nucleus and avoiding the production of classical pro-inflammatory cytokines such as TNF-α, IL11, the colony stimulating factor (CSF), and the Macrophage-derived Oncostatin M (OSM). At endothelial level, BOSB intake increased IL25 production, which lowers the expression of the tumor necrosis factor receptor superfamily TNFRSF14 and the infiltration of monocytes. The potential regulation of neuropeptides by some of the bioactive compounds from BOSB, was reflected in upregulation of ghrelin (GHRL) and downregulation of the thyroid stimulating hormone beta (TSHB). Most of the regulated proteins, converged in pathways that increased mitochondrial fatty acid β-oxidation and reduced acetyl-coA carboxylase enzyme (ACC), producing an overall decrease of the fatty acid synthesis. This negative regulation of the lipid metabolism was expressed in decreased adipogenesis and lipolysis, positively regulating the peroxisome proliferator-activated receptor gamma 2 (PPARγ2).

## Conclusion

Results from this research suggest that the intake of bioactive compounds from BOSB, such as fiber-associated components and polyphenols, attenuates the circulating glucose and TG levels in Mexican women. Among the main metabolic pathways regulated by BOSB consumption, the data driven proteomic analysis showed that HMGB1 and pathways associated with a reduction of local inflammation, inhibition of adipocyte differentiation, increased beta-oxidation of fatty acids, and appetite-linked hormonal regulation promotes the attenuation of hypertriglyceridemia in Mexican women. These results partially validated the inclusion of a functional snack as a healthy strategy for improving the lipid profile and reducing hypertriglyceridemia effects in women. Authors acknowledge that there are some limitations of this study such as the sample size. In general, clinical trials using functional foods have important issues and sometimes it might be more complicated to run than a conventional drug trial for various reasons, including the number of participants, delivery of fresh food to patients, compliance, and free access of participants to other foods (41).

## Data Availability Statement

The original contributions presented in the study are included in the article/Supplementary Material, further inquiries can be directed to the corresponding author/s.

## Ethics Statement

The studies involving human participants were reviewed and approved by the Bioethics Committee of Facultad de Ciencias Naturales, Universidad Autónoma de Querétaro. The patients/participants provided their written informed consent to participate in this study.

## Author Contributions

AR-J: methodology, validation, formal analysis, investigation, data curation, writing-original draft, writing-review and editing, and visualization. IL-O: validation, formal analysis, writing-original daft, writing-review and editing, and visualization. MC-N: validation, formal analysis, writing-original draft, and writing-review and editing. MA-L and ML-G: conceptualization, supervision, and funding acquisition. GL-P: conceptualization, validation, resources, writing-review and editing, supervision, project administration, and funding acquisition. All authors contributed to the article and approved the submitted version.

## Conflict of Interest

The authors declare that the research was conducted in the absence of any commercial or financial relationships that could be construed as a potential conflict of interest.

## Publisher’s Note

All claims expressed in this article are solely those of the authors and do not necessarily represent those of their affiliated organizations, or those of the publisher, the editors and the reviewers. Any product that may be evaluated in this article, or claim that may be made by its manufacturer, is not guaranteed or endorsed by the publisher.
