# Supplementary material for: Daily Intake of a Phaseolus vulgaris L. Snack Bar Attenuates Hypertriglyceridemia and Improves Lipid Metabolism-Associated Plasma Proteins in Mexican Women: A Randomized Clinical Trial
Source: Front Nutr. 2022 Jun 3;9:890136. doi: 10.3389/fnut.2022.890136 (PMC9204147; doi:10.3389/fnut.2022.890136)
Supplement: Supplementary file 1 [file Table_1.DOCX]

**Frontiers in Nutrition**

**Supplementary Electronic Material**

**Daily intake of a common bean and oat bar attenuates hypertriglyceridemia and improves lipid metabolism-associated plasma proteins in Mexican women: a randomized clinical trial**

**Supplementary Table S1.** Differentially expressed proteins after BOSB treatment.

| **Protein** | **Acronym** | **Official symbol** | **Log 2 (Differential expression ratio)** | **p-value** |
| --- | --- | --- | --- | --- |
| Angiotensin-converting enzyme I | ACE/CD43 | ACE | 2.53 | **0.0097** |
| Angiotensin converting enzyme II | ACE-2 | ACE2 | -1.41 | 0.2635 |
| Angiopoietin similar factor | Ang-like factor | ANGPTL | -2.00 | 0.8161 |
| Bone morphogenetic protein 3 | BMP-3 | BMP3 | -1.49 | 0.6484 |
| Growth differentiation factor | BMP-3b/GDF-10 | GDF10 | -2.18 | 0.0524 |
| Bone morphogenetic protein 7 | BMP-7 | BMP7 | -2.67 | 0.2517 |
| Bone morphogenetic protein receptor 2 | BMPR-II | BMPR2 | -1.40 | 0.6205 |
| Nerve growth factor b | b-NGF | NGF | -2.38 | 0.3229 |
| TNF superfamily receptor, member 9 | CD137  (4-1BB) | TNFRSF9 | 1.73 | 0.0802 |
| TYRO3 Protein tyrosine kinase | Dtk | TYRO3 | -1.86 | 0.0537 |
| Epidermal growth factor receptor | EGF-R | EGFR | -1.63 | 0.2984 |
| Fibroblast growth factor 10 | FGF-10 | FGF10 | -2.73 | 0.8069 |
| Growth Hormone 1 | GH | GH1 | -1.29 | 0.0850 |
| Ghrelin/Obestatin | Ghrelin | GHRL | 2.06 | 0.2350 |
| Glucose Transporter Member 2 | Glut2 | SLC2A2 | -1.32 | 0.4699 |
| Interleukin 11 | IL-11 | IL11 | 1.69 | 0.1640 |
| Interleukin 25 | IL-25/IL-17E | IL25 | 1.12 | 0.7129 |
| Leukemia Inhibitory Factor | LIF | LIF | 1.27 | 0.5624 |
| Macrophage stimulating colony factor 1 | M-CSF | CSF1 | -1.75 | 0.1976 |
| Chemokine 3 ligand (C-C motif) | MIP-1a | CCL3 | -2.43 | 0.1448 |
| Neuropilin 1 | Neuropilin-1 | NRP1 | 1.59 | 0.7632 |
| Hypocretin (Orexin) | Orexin A | HCRT | 1.40 | 0.5533 |
| Oncostatin M | OSM | OSM | -1.79 | 0.5099 |
| Osteonectin | Osteonectin | SPARC | -3.30 | **0.0409** |
| Platelet-derived growth factor C | PDGF-C | PDGFC | -1.64 | 0.5118 |
| Delta-like 1 homolog (Drosophila)/Preadipocyte factor | Pref-1 | DLK1 | 2.18 | 0.0739 |
| Prohibitin | Prohibitin | PHB | 1.21 | 0.2088 |
| Retinol-binding protein 4 | RBP4 | RBP4 | -1.46 | 0.1189 |
| Resistin-like b | RELMb | RETNLB | -1.40 | 0.2802 |
| 5-hydroxytryptamine oxigenase regulator (serotonin) | Serotonin | HTOR | -1.90 | **0.0146** |
| Transforming growth factor b | TGF-b | TGFB1 | -1.48 | 0.4970 |
| Thrombospondin 1 | Thrombospondin 1 | THBS1 | -1.92 | 0.2536 |
| TIMP metallopeptidase inhibitor 1 | TIMP-1 | TIMP1 | -1.74 | **0.0081** |
| TIMP metallopeptidase inhibitor 2 | TIMP-2 | TIMP2 | -2.08 | 0.2193 |
| Coagulation Factor 3 (Thromboplastin, tissue factor) | Tissue Factor (CD142) | F3 | 2.75 | 0.1021 |
| Toll-like receptor 2 | TLR2 | TLR2 | 1.43 | 0.3607 |
| TNF superfamily receptor, member 1B | TNF sRII | TNFRSF1B | 1.73 | 0.3225 |
| Thyroid stimulating hormone b | TSH | TSHB | -3.47 | 0.0744 |
| Vascular cell adhesion molecule 1 | VCAM1 | VCAM1 | -1.67 | **0.0660** |

**Supplementary Table S2.** Involved proteins by canonical pathways and physiological functions

| **Classification** | **Involved Proteins** |
| --- | --- |
| **Canonical pathways** | |
| HMGB1 signaling pathway | IL11, IL25, LIF, OSM, TGFB1, TNFRSF1B, VCAM |
| Pathogen-recognition receptor activation | IL11, IL25, LIF, OSM, TGRB1, TLR2 |
| Atherosclerosis signaling pathways | CSF1, F3, PDGFC, RBP4, TGFB1, VCAM1 |
| NF-κB signaling pathway | BMPR2, EGFR, GH1, NGF, TLR2, TNFRSF1B |
| Adipogenesis pathway | BMP7, BMPR2, DLK1, TGFB1 |
| **Physiological functions** | |
| Tissue Morphology | BMP7, BMPR2, CCL3, CSF1, DLK1, EGFR, F3, FGF10, GH1, GHRL, IL11, IL25, LIF, NGF, NRP1, OSM, PDGFC, SPARC, TGFB1, THBS1, TIMP1, TIMP2, TLR2, TNFRSF1B, TYRO3, VCAM1 |
| Cardiovascular system, development, and function | BMP7, BMPR2, CSF1, EGFR, F3, FGF10, GHRL, NGR, NRP1, OSM, PDGFC, RBP4, SPARC, TGFB1, THBS1, TIMP2, VCAM1 |
| Inflammatory response | ACE, CCL3, CSF1, EGF4, GHRL, IL11, IL25, LIF, NGF, OSM, RETNLB, TGFB1, THBS1, TLR2, TNFRSF1B, TNFRSF9, TYRO3, VCAM1 |
| Immune cells trafficking | BMPR2, CCL3, CSF1, EGFR, F3, IL25, OSM, SPARC, TGFB1, THBS1, TIMP1, TLR2, TNFRSF1B, TNFRSF9, VCAM1 |

**Supplementary Figure S1.** Formats with information regarding a) dietary guidelines, b) diet equivalences, c) a food registration diary, and d-e) Adherence to treatment formats that were provided to participants when the study began.


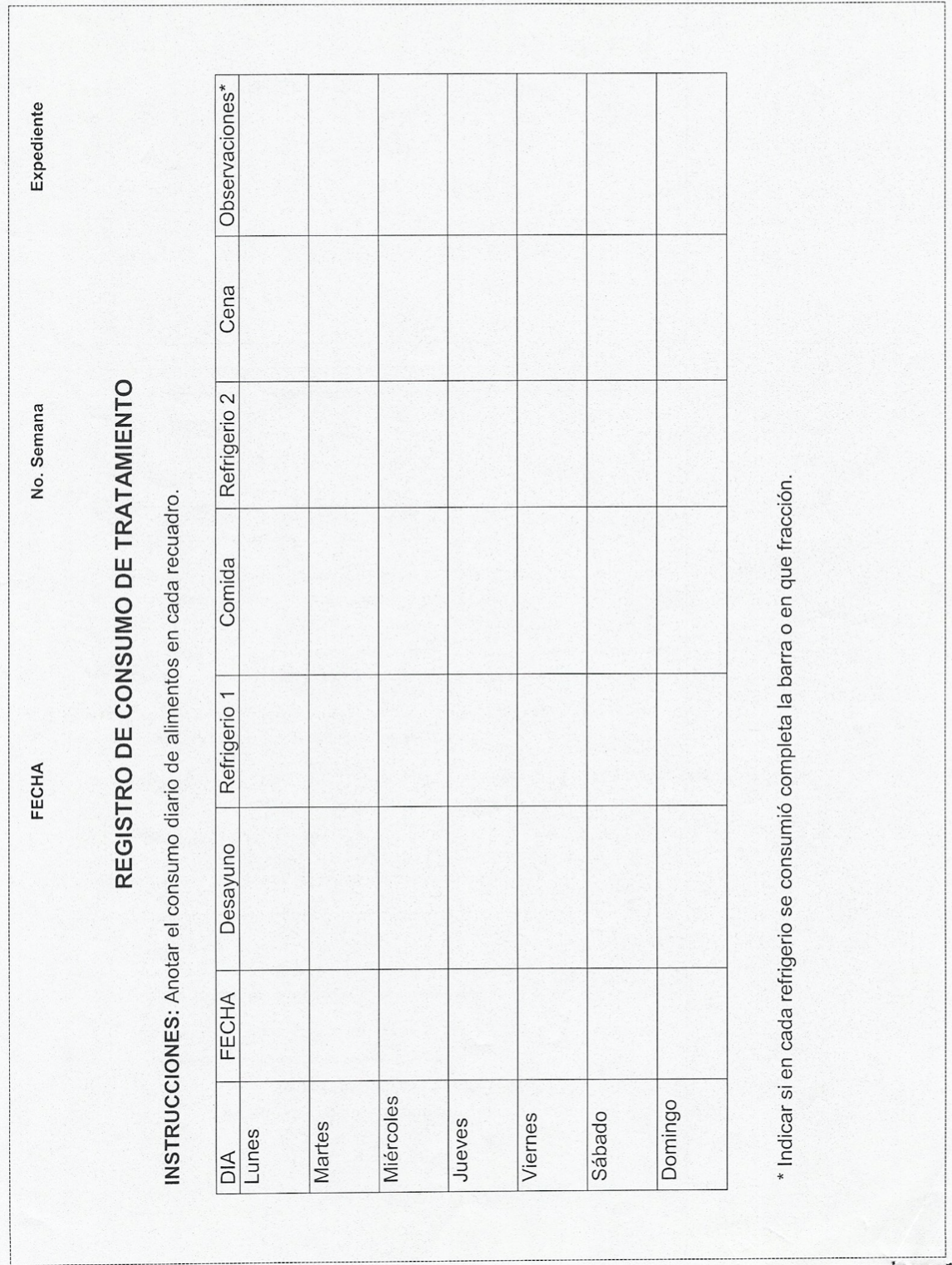

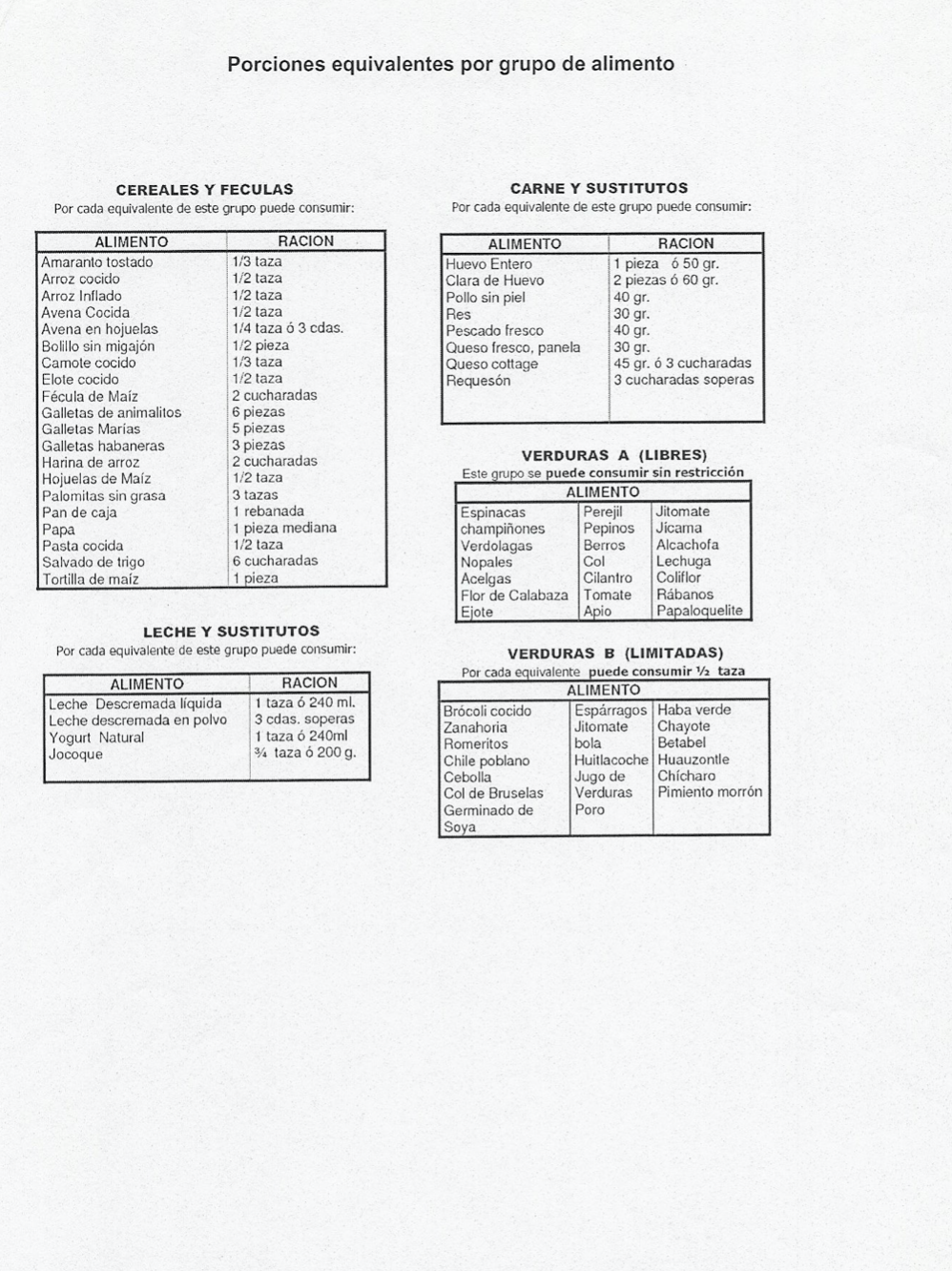

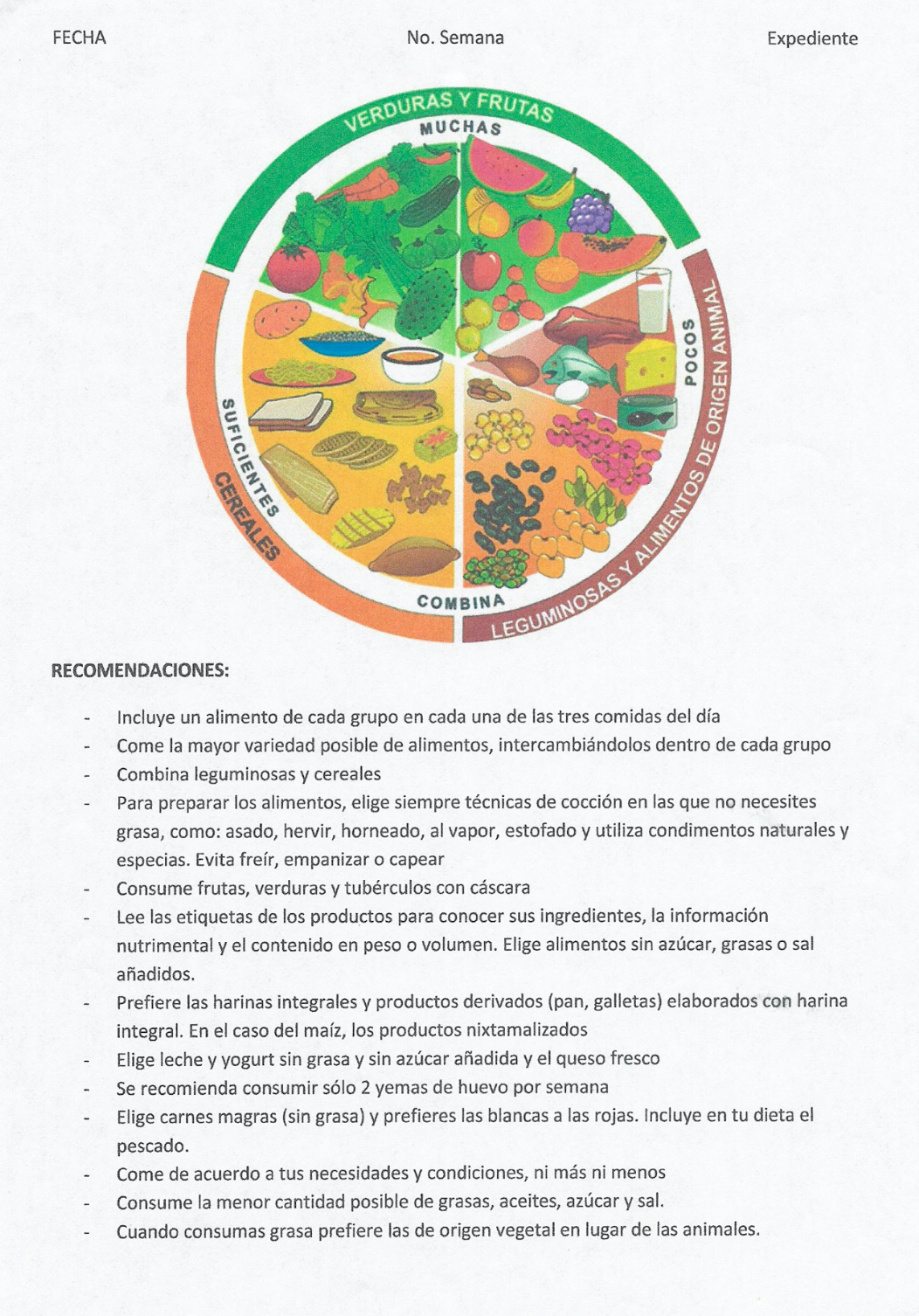


**c)****)**

**b)**

**a)**


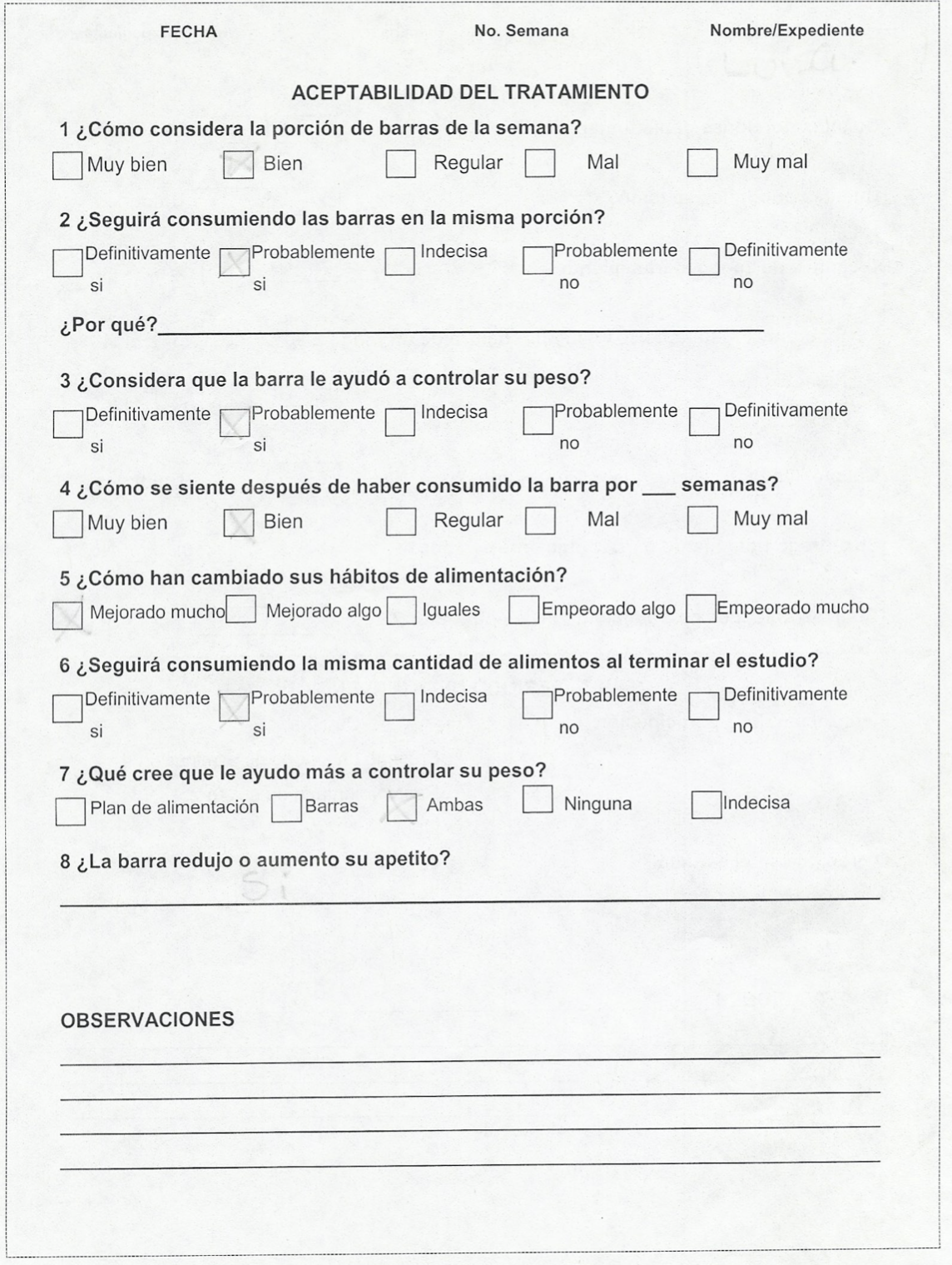

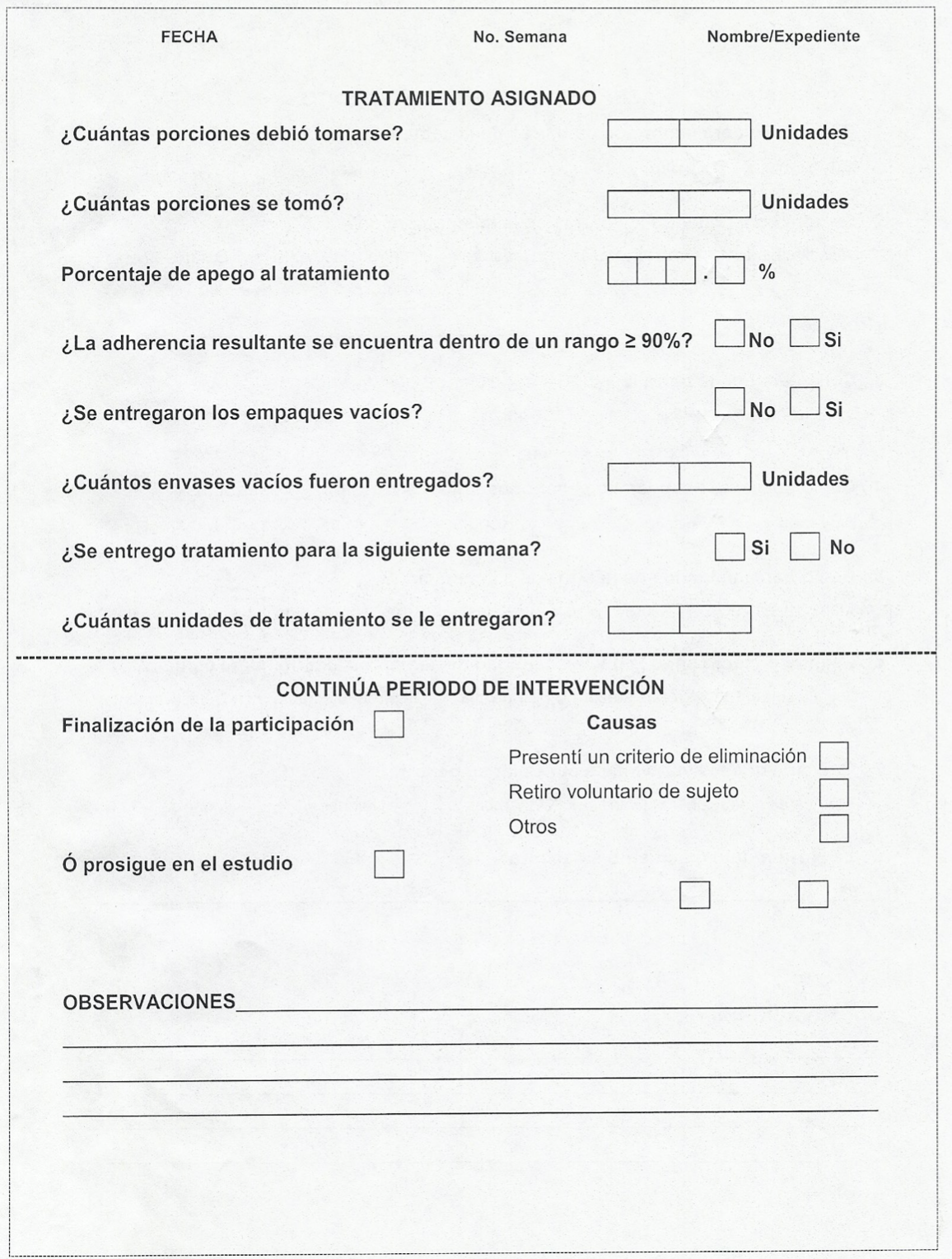


**e)**

**d)**
